# Rapamycin Promotes the Expansion of Myeloid Cells by Increasing G-CSF Expression in Mesenchymal Stem Cells

**DOI:** 10.3389/fcell.2022.779159

**Published:** 2022-03-17

**Authors:** Minghao Li, Yanjie Lan, Juan Gao, Shengnan Yuan, Shuaibing Hou, Tengxiao Guo, Fei Zhao, Yuxia Wang, Weiping Yuan, Xiaomin Wang

**Affiliations:** ^1^ State Key Laboratory of Experimental Hematology, National Clinical Research Center for Blood Diseases, Haihe Laboratory of Cell Ecosystem, Institute of Hematology and Blood Diseases Hospital, Chinese Academy of Medical Sciences and Peking Union Medical College, Tianjin, China; ^2^ Shanghai Blood Center, Shanghai, China; ^3^ Tianjin Eye Hospital, Tianjin Key Laboratory of Ophthalmology and Visual Science, Tianjin Eye Institute, Clinical College of Ophthalmology, Tianjin Medical University, Nankai University Affiliated Eye Hospital, Tianjin, China; ^4^ Department of Neuro-oncology, Cancer Center, Beijing Tiantan Hospital, Capital Medical University, Beijing, China

**Keywords:** rapamycin, mTOR, G-CSF, hematopoiesis, myeloid cells

## Abstract

Rapamycin, also known as sirolimus, an inhibitor of mammalian target of rapamycin (mTOR), is a regulatory kinase responsible for multiple signal transduction pathways. Although rapamycin has been widely used in treating various hematologic diseases, the effects of rapamycin are still not fully understood. Here we found that both oral and intraperitoneal administration of rapamycin led to the expansion of myeloid lineage, while intraperitoneal administration of rapamycin impaired granulocyte differentiation in mice. Rapamycin induced bone marrow mesenchymal stem cells to produce more G-CSF *in vitro* and *in vivo*, and promoted the myeloid cells expansion. Our results thus demonstrated that intraperitoneal administration of rapamycin might promote the expansion of myeloid lineage while impair myeloid cell differentiation *in vivo*.

## Introduction

Rapamycin (Sirolimus), initially discovered as an antifungal metabolite, produced by *Streptomyces hygroscopicus* from a soil sample of Easter Island (also known as Rapa Nui) (Vézina et al., 1975). It has been reported that rapamycin binds to the immunophilin FK506 binding protein-12 (FKBP12) and inhibits the activation of the mammalian target of rapamycin (mTOR). PI3K-AKT-mTOR pathway was constitutively activated in 60% of AML patients, and the over-activation of PI3K-AKT-mTOR was associated with poor survival of AML patients (Min et al., 2003; Kornblau et al., 2009; Chen et al., 2010; Nepstad et al., 2018; Nepstad et al., 2019). Targeted deletion of mTORC1 in mouse models enhances HSC self-renewal and repopulating properties, and highlights the importance of the individual mTOR-containing complexes at specific stages of HSC homeostasis and haemopoietic lineage commitment and maturation (Malik et al., 2018). Rapamycin, through its inhibition of mTOR, enhanced the anti-tumor effects of doxorubicin on CML cells (Li et al., 2019). *In vitro*, rapamycin promoted CML cells apoptosis and inhibited CML cell cycle through blocking mTOR signaling pathway (Li et al., 2019). Moreover, rapamycin has been tested alone or in combination with Janus kinase (JAK), ABL protein inhibitors (Tasian et al., 2017), focal adhesion kinase (FAK) or also with cyclin D3 (CCND3) and CDK4/6 inhibitors (Li et al., 2015; Pikman et al., 2017) in xenografts mouse model and cancer cell lines, exhibiting synergistic effects on anti-tumor. Although rapamycin has been developed as a second-generation immunosuppressive and anti-proliferative agent for tumor treatment, the functional impact of rapamycin treatment as an inhibitor of mTORC1 pathway on normal hematopoiesis was not fully understood.

Currently, rapamycin is in early phase clinical trials for a number of malignancies based on the importance of the AKT-mTOR pathway in cancer biology. It has been reported that rapamycin could increase doxorubicin-induced apoptosis of mononuclear cells from non-responder childhood acute lymphoblastic leukemia patients (Avellino et al., 2005) and augment cell sensibility to glucocorticoid induced apoptosis in a subset of primary ALL patients (Wei et al., 2006). The efficacy of rapamycin is also under evaluation in combination with Donor Stem Cell Transplant in adult Ph + B-ALL patients (see www.clinicaltrials.gov/NCT00792948). Despite rapamycin inhibits leukemia cell growth and enhances the anti-tumor effects of chemotherapeutic agents, the usage of rapamycin is limited by its side effects. The intraperitoneal administration of rapamycin induced gastrointestinal discomfort and mouth ulcers, impaired wound healing and elevated circulating triglycerides (Augustine et al., 2007; de Oliveira et al., 2011). However, many of these effects/side effects have not been observed or fully investigated at lower doses of rapamycin in mice (Zhang et al., 2012; Siegmund et al., 2017) or human (Kraig et al., 2018; Yoon et al., 2018). Before rapamycin can be used in leukemia patients, it is critical to understand the undesirable side effects of rapamycin and define a suitable administration of rapamycin to provide its highest benefit while limiting the side effects.

In this study, we explored the effects/side effects of rapamycin on normal hematopoietic cells via inhibiting mTORC1 signaling pathway. We found that oral and intraperitoneal administration of rapamycin increased myeloid cells proliferation through increasing G-CSF expression in MSCs, while intraperitoneal administration of rapamycin impaired neutrophil differentiation.

## Materials and Methods

### Mice and Genotyping

Rheb1 conditional deletion mice were generated using the homologous recombination technique to flank exon three of Rheb1 with two LoxP sequences (Figure 4A). The mice were then mated with *Lyz-Cre* transgenic mice expressing *Cre* recombinase under the control of the Lyz promoter to delete *Rheb1* in the myeloid cells at the embryonic stage. As expected, half of the offspring were *Rheb1* wild-type (*Rheb1* ^*fl/fl*^), and the other half were *Rheb1* knockout mice (*Lyz-Cre*; *Rheb1* ^*fl/fl*^ or *Rheb1* ^*Δ/Δ*^). Mice were maintained at the specific pathogen-free (SPF) animal facility of the State Key Laboratory of Experimental Hematology (SKLEH). All animal protocols were approved by the Institutional Animal Care and Use Committee (IACUC), the Institute of Hematology, and Blood Diseases Hospital (CAMS/PUMC). All surgery was performed under sodium pentobarbital anesthesia, and every effort was made to minimize mouse suffering.

### Drugs

Rapamycin (LC Lab, USA) was dissolved in ethanol at 10 mg/ml as stock solution. Its injection solution was further dissolved in PBS with PEG-400 and Tween-80 as cosolvent. The wild type (WT) mice received rapamycin or vehicle (as the control) at dose of 4 mg/kg/day by i. p. every other day for 1 month. The recipient mice received rapamycin at dose of 2 mg/kg/day by p. o. for 4 months after transplantation.

### Flow Cytometry Analysis

20 μL peripheral blood (PB) was obtained from either the tail veins or retroorbital bleeding of mice and diluted with PBE (PBS with 2% fetal bovine serum and 2 mM EDTA). Red blood cells (RBCs) were lysed by red blood cells lysis buffer before staining. Bone marrow (BM) cells were flushed out from tibias, femurs, and ilia by PBE. Cells were stained with antibodies purchased from either eBioscience or BD Bioscience. The cells were stained with the following antibodies: anti-mouse CD3 PE-cy7, anti-mouse B220 FITC, anti-mouse CD11b APC, anti-mouse Ly-6G PE-cy7 for analyzing different lineages. Anti-mouse CD3 biotin, anti-mouse Ly-6G biotin, anti-mouse CD11b biotin, anti-mouse TER-119 biotin, anti-human/mouse B220 biotin, streptavidin FITC, anti-mouse CD117 (c-Kit) APC, anti-mouse Ly-6A/E (Sca-1) PE-Cyanine7, anti-mouse CD45.2 Percp-cy5.5, and anti-mouse CD45.1 PE for HSPCs or anti-mouse CD45.1 FITC, anti-mouse CD45.2 PE, anti-mouse CD11b APC, and anti-mouse Ly-6G (Gr-1) PE-Cyanine7 for neutrophils. All antibodies were purchased from either eBioscience or Invitrogen (United States). To analyze intracellular proteins, 3×10^6^ BM cells were labeled with surface antibodies, fixed with 4% paraformaldehyde, permeabilized with 0.1% Triton X100, then washed 2 times with 1 ml cold PBE. Finally, the cells were resuspended with cold PBS supplemented with 25% FBS, and intracellularly stained with anti-bodies: p-S6 (Ser24/244), p-4EBP1 (Thr37/46). Cells were analyzed by BD Canto II flow cytometer. FlowJo software was used to analyze the results.

### Competitive Bone Marrow Transplantation and Analysis

Donor BM cells were isolated from the tibias, femurs and ilia from 8-week-old C57BL/6(B6) mice (CD45.1^+^). 5×10^5^ (CD45.1^+^) together with 5×10^5^ WBMCs (CD45.2^+^) were intravenously injected into the lethally irradiated recipient mice (CD45.2^+^). Then, the reconstituted PB cells were analyzed every 4 weeks after transplantation.

### Quantitative Real-Time PCR (qRT-PCR)

RNA from BM samples was isolated using the RNeasy Mini Kit (QIAGEN, 74106, Germany). cDNA synthesis was performed using a cDNA reverse transcription kit (Takara, RR047A, Japan) according to the manufacturer’s protocol. Quantitative PCR assays were performed in 96-well Micro Amp Fast Optical Reaction Plates (Applied Biosystems, 4344904, United States) using SYBR Green PCR Master Mix (Roche, 04913914001, Switzerland). The signal was detected using the Step-One Plus Real-Time PCR System (QuantStudio5). GAPDH was used as an endogenous control for gene expression assays.

### Isolation of MSCs From Bone and MSCs Culture

Mesenchymal stem cells (MSCs) from the compact bones of mice were obtained as previously described (Zhu et al., 2010). The bone cavities were washed thoroughly at three times using a syringe until the bones become pale to deplete hematopoietic cells from the tibiae and femurs. Hold the humeri, tibiae and femurs with forceps and excise the compact bones carefully into chips of approximately 1–3 mm^3^ with scissors. The bone chips were transferred into a 25-cm^2^ plastic culture flask with forceps, then suspend the chips in 3 ml of α-MEM (Hyclone, SH30265.01, United States) containing 10% FBS (Gibco, 16000-044, United States) in the presence of 1 mg/ml of collagenase II (Gibco, 17101015, United States). The chips were digested for 1–2 h in a shaking incubator at 37°C with a shaking speed of 200 rpm. The collagenase digestion was stopped when the bone chips become loosely attached to each other. The digestion medium and released cells were aspirated and discarded. Enzyme-treated bone chips were placed in a 10 cm^2^ dish containing 6 ml of α-MEM supplemented with 10% FBS. Each replanting was considered a passage. The surfaces marker of MSCs (Lin^-^CD45^−^CD31^−^CD51^+^Sca-1^+^) were analyzed by Flow cytometry.

### Lin^-^ c-kit^+^ (LK^+^) Isolation and Cocultured With MSCs

BM cells were isolated from the tibias, femurs and ilia of 8-week-old B6. SJL mice. WT LK^+^ cells were sorted with a c-Kit (CD117) Microbead Kit (MACS, 130-091-224, German) and a Lineage Cell Depletion Kit (MACS, 130-090-858, German) according to the manufacturer’s protocol. 5×10^4^ MSCs were cultured in 24-well plate in a volume of 800 μL α-MEM with 15% FBS and treat with rapamycin or ethanol. After 24 h of culture, the MSCs were cocultured with 2×10^5^ LK^+^ cells. After 24 h of coculture, the MSCs were harvested. The LK^+^ cells were analyzed for the percentage and absolute number of myeloid cells by flow cytometry. For the G-CSF neutralization experiment, G-CSF antibody (R&D, MAB406-SP, United States) was added to the coculture system at 2 nM. After 24 h of coculture. The LK^+^ cells were analyzed for the percentage and absolute number of myeloid cells by flow cytometry. All cells were incubated at 37°C in a 5% CO_2_ incubator.

### ELISA

The ELISA was performed using the Mouse G-CSF ELISA Kit (Anoric, TAE-317m, China) according to the manufacturer’s protocols. A total of 3×10^5^ MSC in 200 μL of PBS were frozen and thawed three times and centrifuged at 5,915 rpm (3,000 g) for 10 min, and the liquid supernatants were collected for G-CSF determination. The cell culture medium was concentrated with an ultragentrification device (Millipore, UFC801096, Germany). The ELISA tests were read on a SynergyH four Hybrid Reader at 450 nm.

### Statistical Analyses

GraphPad Prism 8.0 was used for statistical analyses. Every experiment was compared as two groups. All results were analyzed using the unpaired two-tailed Student t-test. *p* < 0.05 was considered significant for all tests. All data are presented as the mean ± standard deviation (SD); Significant difference are indicated with asterisks (*p < 0.05; **p < 0.01; ***p < 0.001).

## Results

### Intraperitoneal Administration of Rapamycin Increased Myeloid Cells Proliferation

**FIGURE 1:**
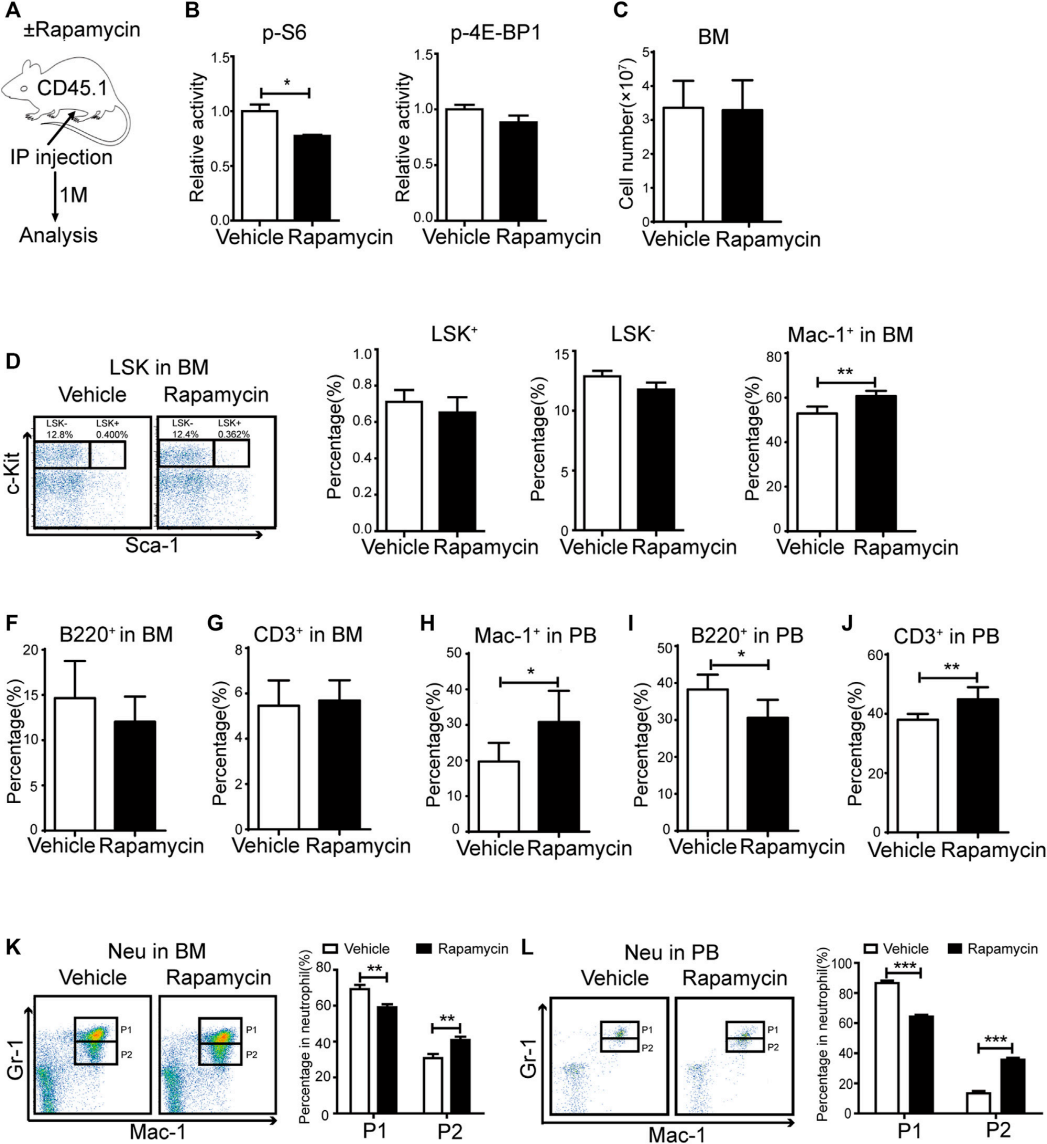
Intraperitoneal administration of rapamycin enhanced myeloid cells proliferation **(A)** Experiment design following rapamycin or vehicle treatment, n = 4, days = 30, dose = 4 mg/kg/day **(B)** Fluorescence intensity of p-S6 and p-4E-BP1 in bone marrow **(C)** Absolute number of bone marrow cells **(D)** FACS analysis of LSK^+^ (Lin^-^c-Kit^+^Sca-1^+^) and LSK^-^ (Lin^-^c-Kit^+^Sca-1^-^) cells in BM (left panel). Percentage of LSK^+^ and LSK^-^ in BM of mice that injected with rapamycin or vehicle (right panel) **(E–G)** Percentage of myeloid cells (CD11b^+^), B cells (B220^+^), T cells (CD3^+^) by FACS in BM in two groups **(H–J)** Percentage of myeloid cells (CD11b^+^), B cells (B220^+^), T cells (CD3^+^) by FACS in PB in two groups **(K)** FACS analysis of neutrophils in BM of mice treated by rapamycin or vehicle, n = 4 (left panel). Percentage of neutrophil subpopulations in two groups; n = 4 (right panel) **(L)** FACS analysis of neutrophils in PB of mice treated by rapamycin or vehicle, n = 4 (left panel). Percentage of neutrophil subpopulations in two groups, n = 4 (right panel).

To evaluate the effects of rapamycin in hematopoietic cells, we injected wild type (WT) mice with rapamycin (4 mg/kg/d) or vehicle by i. p. every other day for 1 month and analyzed hematopoietic cell populations in bone marrow (BM) and peripheral blood (PB) by flow cytometry (FACS) (Figure 1A). As mTORC1 effectors, the phosphorylation status of S6 and 4 E-BP1 in BM cells was quantitatively evaluated by flow cytometry (FACS) using intracellular staining with antibodies against p-S6 (S240/244) and p-4E-BP1 (T37/46). The fluorescence intensity of p-S6 was decreased in BM cells of mice treated with rapamycin when compared that of controls, while the fluorescence intensity of p-4E-BP1 was not changed in BM cells of mice treated with rapamycin (Figure 1B). BM cells and PB cells were collected from mice at 4 weeks after treatment with rapamycin by i. p. and were stained with antibodies to detect hematopoietic stem cells (LKS^+^, Lin^-^ c-Kit^+^ sca1^+^), hematopoietic progenitor cells (LKS^-^, Lin^-^ c-Kit^+^ sca1^-^), B cells (B220^+^), T cells (CD3^+^) and Myeloid cells (Mac-1^+^). The absolute number of BM cells in mice treated with rapamycin was equivalent to that of control mice (Figure 1C). The percentage of LKS^+^ and LKS^-^ cells in mice treated with rapamycin was not altered when compared with controls (Figure 1D). The percentage of myeloid cells was increased in BM of mice injected with rapamycin, while the percentage of B cells and T cells was not changed in two groups (Figure 1E–G). In consistent with the increase of myeloid cells in BM, the percentage of myeloid cells was also expanded in PB of mice treated with rapamycin (Figure 1H). The proportion of B cells in PB of mice injected with rapamycin was slightly decreased contrast with that of control mice (Figure 1I), while the percentage of T cells in PB was increased (Figure 1J). We further analyzed characteristics of neutrophils by FACS with CD11b and Ly-6G antibodies that have been used as neutrophil subpopulation markers for the identification of myelocytes/promyelocytes, as well as immature and mature neutrophils (Wang et al., 2019). We divided neutrophils into two distinct subpopulations, indicated as the CD11b^+^ Ly-6G^high^ population (P1) and the CD11b^+^Ly-6G^low^ population (P2). We found that the percentage of the P2 sub-population was increased, while the percentage of the P1 sub-population was decreased both in the BM and PB of mice treated with rapamycin (Figure 1K, L). These data demonstrated that intraperitoneal administration of rapamycin could increase myeloid cells proliferation and impair neutrophil development *in vivo*.

### Oral Administration of Rapamycin Enhanced BM Cells Engraftment Upon Transplantation

**FIGURE 2:**
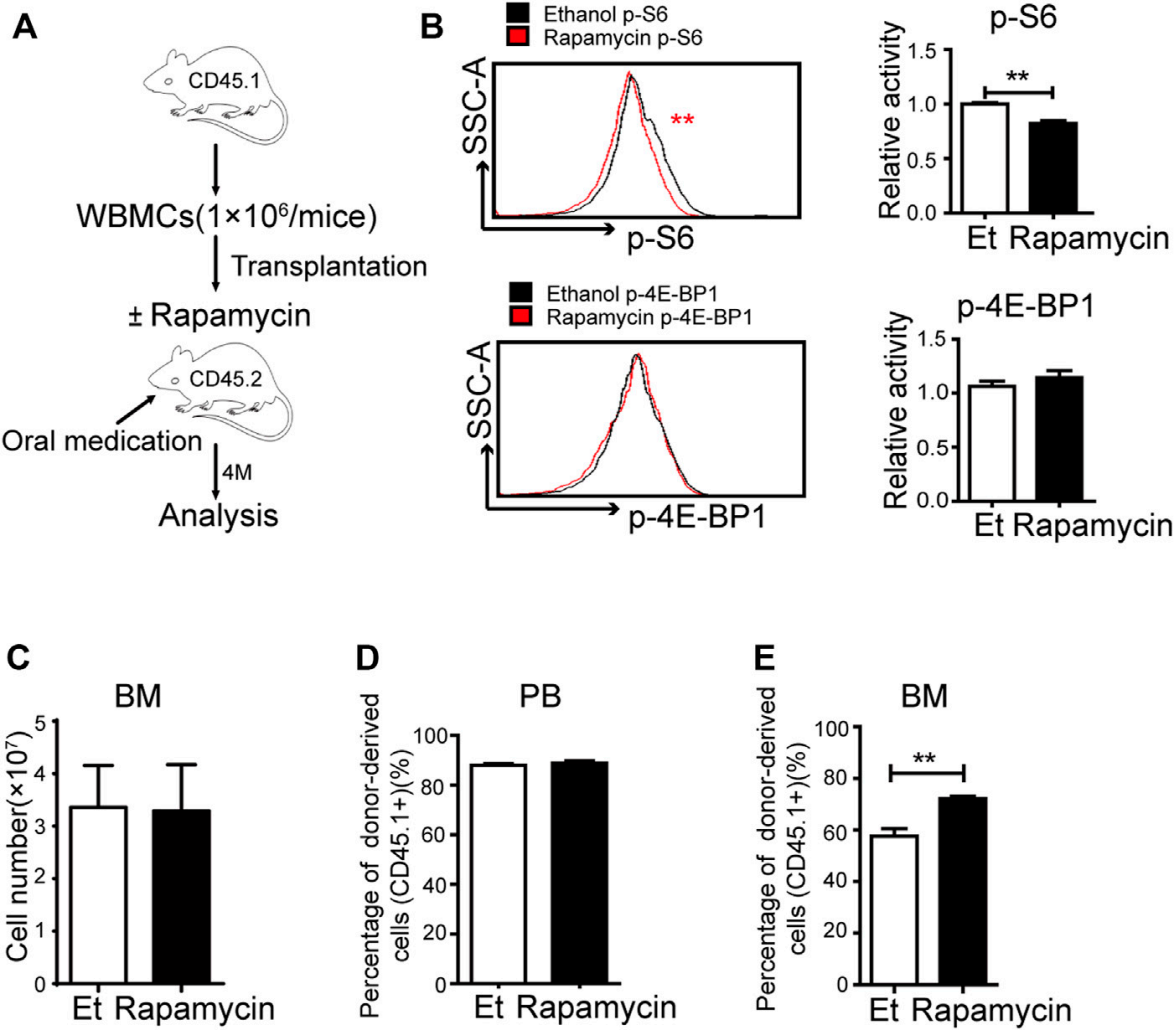
Oral administration of rapamycin enhanced BM cells engraftment upon transplantation **(A)** Experiment design with rapamycin treatment of transplantation mouse model with BM cells (CD45.1), n = 3, days = 120, dose = 2 mg/kg/day **(B)** Fluorescence intensity of p-S6 (upper panel) and p-4E-BP1 (lower panel) in BM cells of recipient mice treated by rapamycin or ethanol (control, Et) for 4 months, n = 3 **(C)** Absolute number of bone marrow cells **(D)** Percentage of donor-derived cells (CD45.1^+^) in the PB in two groups at 4 months; n = 3 **(E)** Percentage of donor-derived cells in the BM in two groups at 4 months; n = 3.

To investigate the efficacy of rapamycin in hematopoietic regeneration during transplantation, we transplanted BM cells (CD45.1^+^) to lethally irradiated mice (CD45.2^+^) and treated these mice with rapamycin or vehicle by p. o. for 4 months (Figure 2A). The recipient mice were sacrificed at 4 months after transplantation and BM cells (CD45.1^+^) were analyzed. The phosphorylation status of S6 was lower in BM cells of recipient mice treated with rapamycin by p. o. than that of control mice (Figure 2B, up panel). The fluorescence status of p-4E-BP1 in BM cells of recipient mice treated with rapamycin by p. o. was equivalent to that of control mice (Figure 2B, down panel). We next analyzed the regeneration ability of BM cells and found that the absolute number of BM cells of mice treated with rapamycin by p. o. was equivalent to that of controls (Figure 2C). The percentage of donor-derived cells was more than 80% in PB of recipient mice with rapamycin or vehicle by p. o. for 4 months, which indicated the hematopoietic system was recovered by donor BM cells (CD45.1^+^) in two groups (Figure 2D). Notably, the percentage of donor-derived cells in BM of recipient mice treated with rapamycin by p. o. was significantly increased compared with that of controls (Figure 2E). It suggested that oral administration of rapamycin could improve regenerated ability of hematopoietic cells upon transplantation.

### Oral Administration of Rapamycin Increased Myeloid Cells Proliferation Upon Transplantation

**FIGURE 3:**
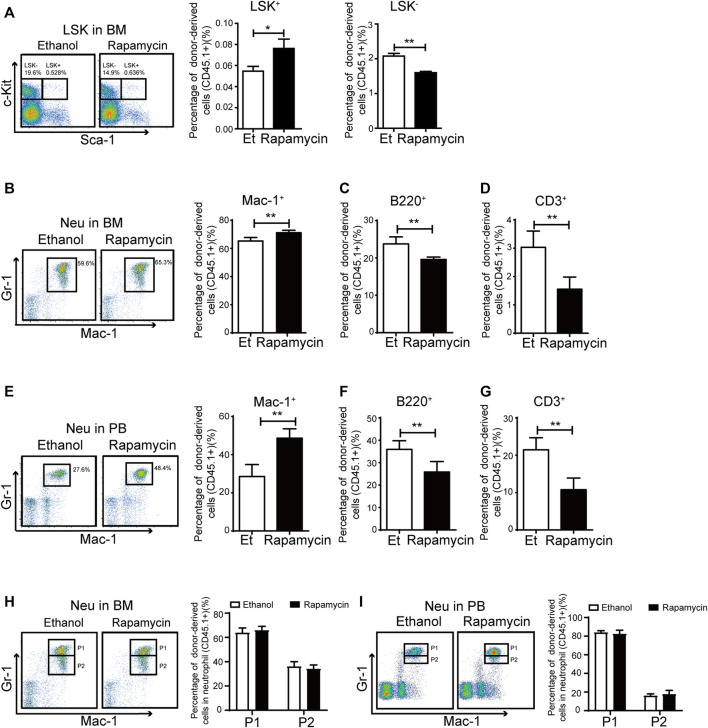
Oral administration of rapamycin enhanced myeloid cells proliferation upon transplantation **(A)** Percentage of donor-derived LKS^+^ (CD45.1) and donor-derived LKS^–^ (CD45.1) cells in BM of mice (CD45.2) treated by rapamycin or ethanol for 4 months, n = 3 **(B–D)** Percentage of donor-derived myeloid cells (CD45.1), donor-derived B cells (CD45.1) and donor-derived T cells (CD45.1) in BM of two groups, n = 3 **(E–G)** Percentage of donor-derived myeloid cells (CD45.1), donor-derived B cells (CD45.1) and donor-derived T cells (CD45.1) in PB cells of two groups, n = 3 **(H–I)** FACS analysis of donor-derived neutrophils (CD45.1) in BM and PB of mice treated with rapamycin or ethanol for 4 mouths, n = 3.

To clarify whether oral administration of rapamycin has certain effects in hematopoietic cells upon transplantation, we analyzed the proportion of hematopoietic stem/progenitor (HSC/HPCs) populations and several mature populations in BM of recipient mice (CD45.2^+^) treated with rapamycin or vehicle by p. o. (Figure 2A). The percentage of donor-derived LSK^+^ cells in BM of recipient mice treated with rapamycin by p. o. was equivalent to that of controls (Figure 3A, middle panel). However, the percentage of LSK^-^ cells was decreased in BM of recipient mice treated with rapamycin when compared with that of controls (Figure 3A, right panel). The percentage of donor-derived myeloid cells was significantly increased, while the percentage of donor-derived T cells and B cells was decreased in BM of recipient mice treated with rapamycin by p. o. when compared with that of controls (Figure 3B–D). In consistent with this, the proportion of donor-derived myeloid cells was also increased in PB of recipient mice treated with rapamycin by p. o. for 4 months (Figure 3E). The percentage of donor-derived T cells and B cells was decreased in PB of recipient mice treated with rapamycin by p. o. when compared with that of controls (Figure 3F,G). Interestingly, the percentage of donor-derived neutrophil P1 sub-population and P2 sub-population was not changed in BM and PB of recipient mice treated with rapamycin by p. o. for 4 months (Figure 3H,I). These results indicated that oral administration of rapamycin induced expansion of myeloid cells, but not influenced the development of neutrophils upon transplantation.

### Inhibiting mTORC1 Signaling didn’t Influence Myeloid Cells Proliferation and Differentiation

**FIGURE 4:**
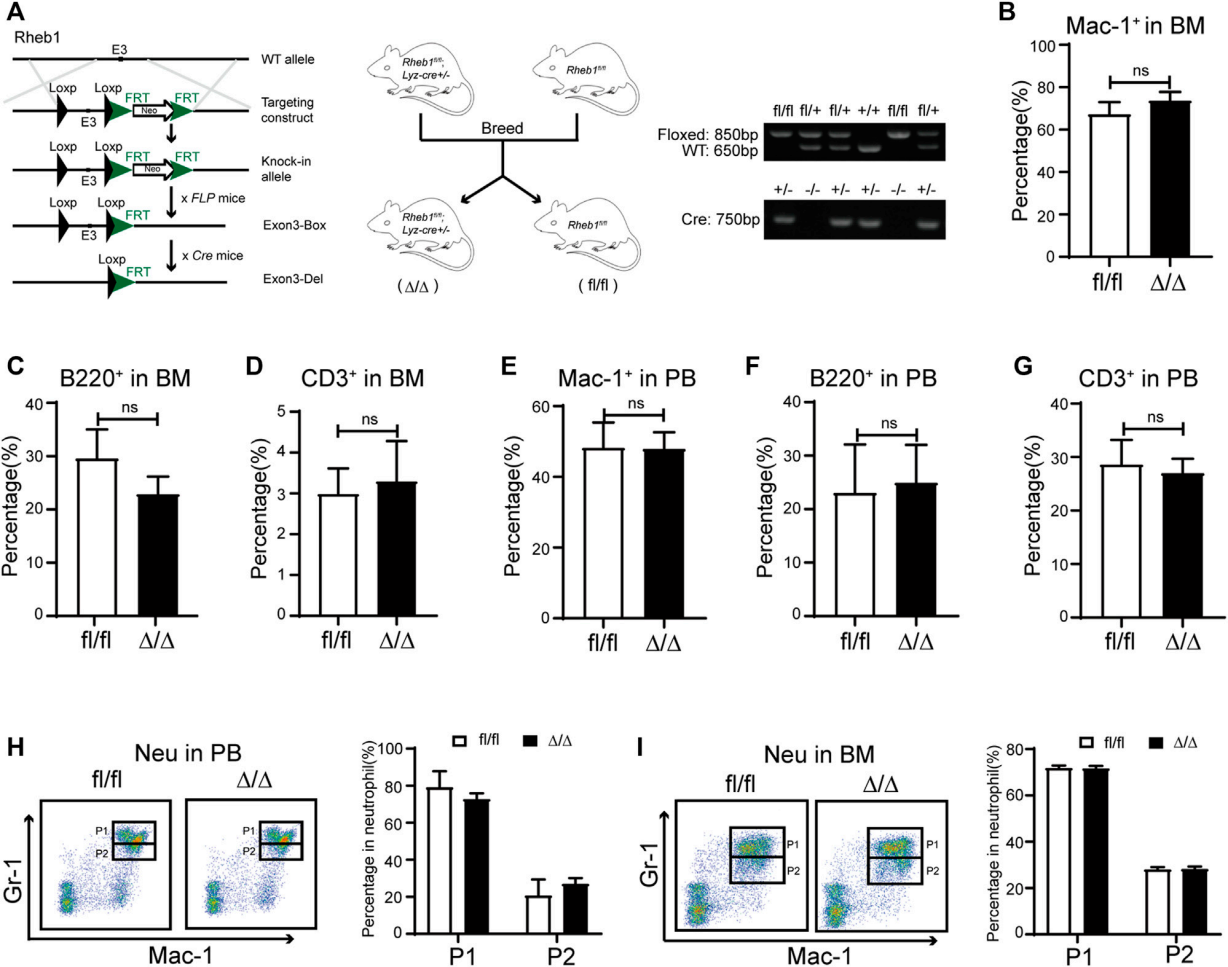
Inhibiting mTORC1 signaling in myeloid cells didn’t influence its proliferation and differentiation **(A)** Mice model of *Lyz-cre; Rheb1* ^*fl/fl*^ mice (*Rheb1* ^*Δ/Δ*^), specific deletion of Rheb1 in the myeloid cells (left and middle panel). The offspring were identified by PCR and analyzed by agarose gel. The size of floxed fragment was 850bp while wild type was 650bp, and the 750 bp segment was amplified from *Cre* ^*+/-*^ (right panel) **(B–D)** Percentage of B cells, T cells and myeloid cells in BM of *Rheb1* ^*fl/fl*^ and *Rheb1* ^*Δ/Δ*^; n = 4 **(E–G)** Percentage of B cells, T cells and myeloid cells in PB of *Rheb1* ^*fl/fl*^ and *Rheb1* ^*Δ/Δ*^; n = 4 **(H)** FACS analysis of neutrophils in PB of *Rheb1* ^*fl/fl*^ and *Rheb1* ^*Δ/Δ*^, n = 4 **(I)** FACS analysis of neutrophils in BM of *Rheb1* ^*fl/fl*^ and *Rheb1* ^*Δ/Δ*^, n = 4.

To investigate whether rapamycin impaired neutrophil differentiation through inhibiting mTORC1 signaling pathway, we bred *Lyz-cre; Rheb1* ^*fl/fl*^ mice (*Rheb1* ^*Δ/Δ*^), in which Rheb1 was deleted and mTORC1 signaling pathway was inhibited specifically in the myeloid cells. The genotype of *Rheb1* ^*fl/fl*^ and *Lyz-cre; Rheb1* ^*fl/fl*^ mice was verified by genomic PCR (Figure 4A). We found the percentage of myeloid cells, T cells and B cells in BM and PB of *Rheb1* ^*Δ/Δ*^ mice was equivalent to that of *Rheb1* ^*fl/fl*^ mice (Figure 4B–G). The percentage of neutrophil P1 sub-population and P2 sub-population was not changed in BM and PB of *Rheb1* ^*Δ/Δ*^ mice when compared with that of *Rheb1* ^*fl/fl*^ mice (Figure 4H,I). These data indicated that inhibiting mTORC1 signaling pathway did not influence proliferation and differentiation of myeloid cells.

### Rapamycin Increased G-CSF Expression in MSCs *in vitro*

**FIGURE 5:**
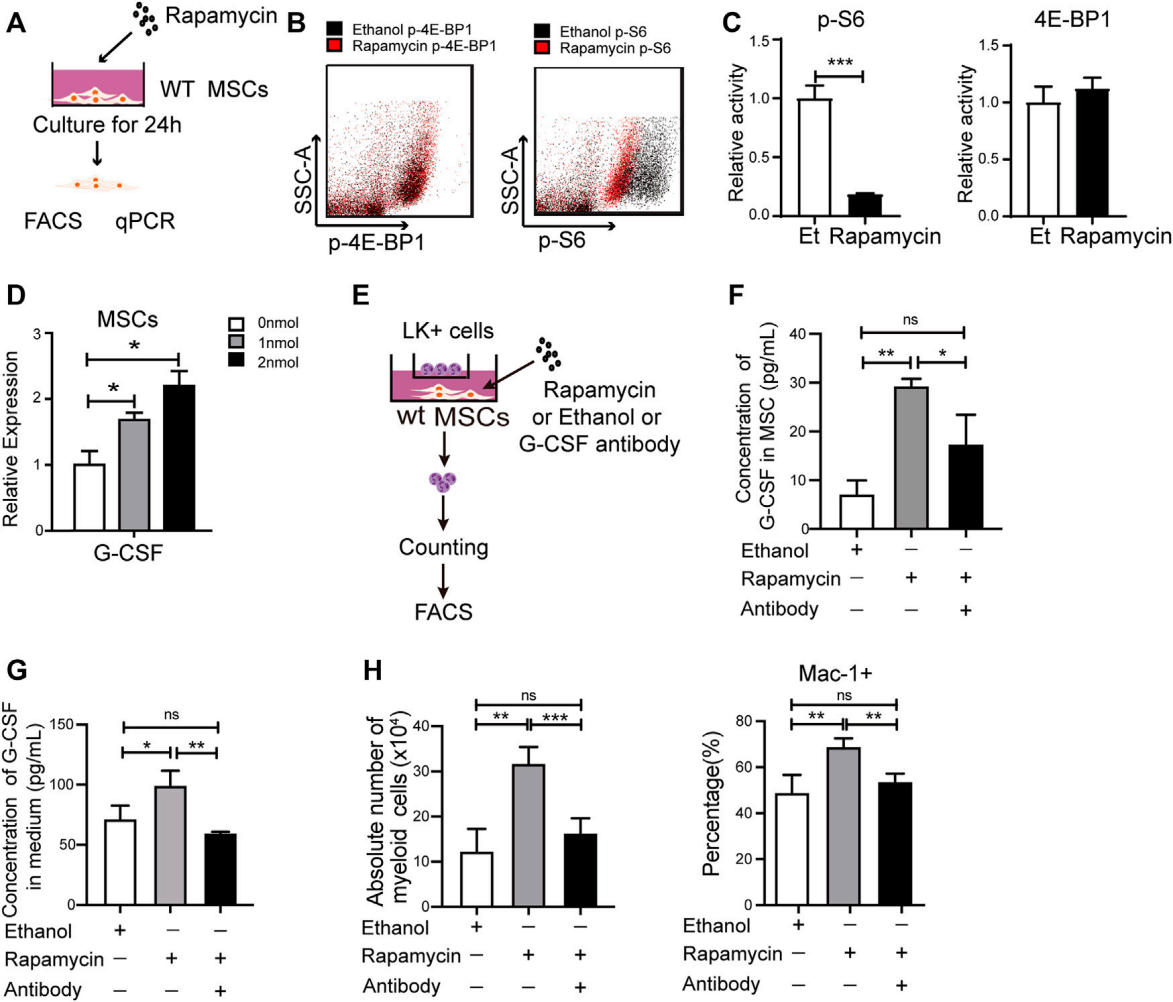
Rapamycin increased G-CSF expression in MSCs *in vitro* **(A)** Experiment design for MSCs treated by rapamycin *in vitro* **(B–C)** Fluorescence intensity of p-S6 and p-4E-BP1 in MSCs treated by rapamycin or ethanol *in vitro* **(D)** mRNA expression of G-CSF in MSCs treated by rapamycin or ethanol *in vitro* **(E)** Experiment design for the co-culture of MSC and LK^+^ cells **(F)** The protein levels of G-CSF in the cell lysates of MSC treated by rapamycin or ethanol **(G)** The G-CSF level in the media of MSC treated by rapamycin or ethanol **(H)** The percentage (Left panel) and absolute number (right panel) of myeloid cells after coculturing with MSCs treated by rapamycin + G-CSF antibody, singel rapamycin, or ethanol.

G-CSF plays an essential role in myeloid expansion and is mainly secreted by Mesenchymal Stem Cells (MSCs) in BM (Lieschke et al., 1994; Boettcher et al., 2014; Lin et al., 2020). To better understand the underlying mechanisms that rapamycin increased expansion of myeloid cells, we isolated MSCs from the BM and cultured MSCs with rapamycin or ethanol treatment for 24 h *in vitro* (Figure 5A). We found the phosphorylation of S6 was significantly reduced, while the phosphorylation of 4 E-BP1 was not changed in MSCs treated with rapamycin when compared with that of MSCs treated with ethanol (Figure 5B,C). Furthermore, we cultured MSCs with different concentrations of rapamycin and measured the mRNA expression of G-CSF in MSCs by qPCR. We found the mRNA expression of G-CSF was increased sequentially in MSCs after treatment with various doses of rapamycin (Figure 5D). We further cocultured 2×10^5^ LK^+^ cells with 5 ×10^4^ MSCs after treatment with rapamycin or ethanol (Figure 5E). We measured the protein expression of G-CSF in the cell lysates of MSC and coculture medium. We found the G-CSF was increased in the MSC treated with rapamycin when compared with that in MSC treated with ethanol (Figure 5F). The secreted G-CSF was also higher in medium of MSC treated by rapamycin than that of MSC treated by ethanol (Figure 5G). In addition, we found that the percentage and absolute number of myeloid cells was much higher in MSC treated by rapamycin than that in MSC treated by ethanol (Figure 5H). Furthermore, we blocked G-CSF by adding G-CSF neutralizing antibody in the media in which MSCs treated with rapamycin for 24 h and cocultured with LK^+^ cells. The G-CSF level in the MSC and medium was decreased after adding G-CSF neutralizing antibody when compared with that treated by rapamycin alone (Figure 5F,G). We found that blockage of G-CSF significantly decreased the percentage and absolute number of myeloid cells than control (Figure 5H). These data suggested that rapamycin might promote myeloid cells expansion by stimulating MSCs to produce G-CSF directly *in vitro*.

### Rapamycin Increased G-CSF Expression in MSCs *in vivo*

**FIGURE 6:**
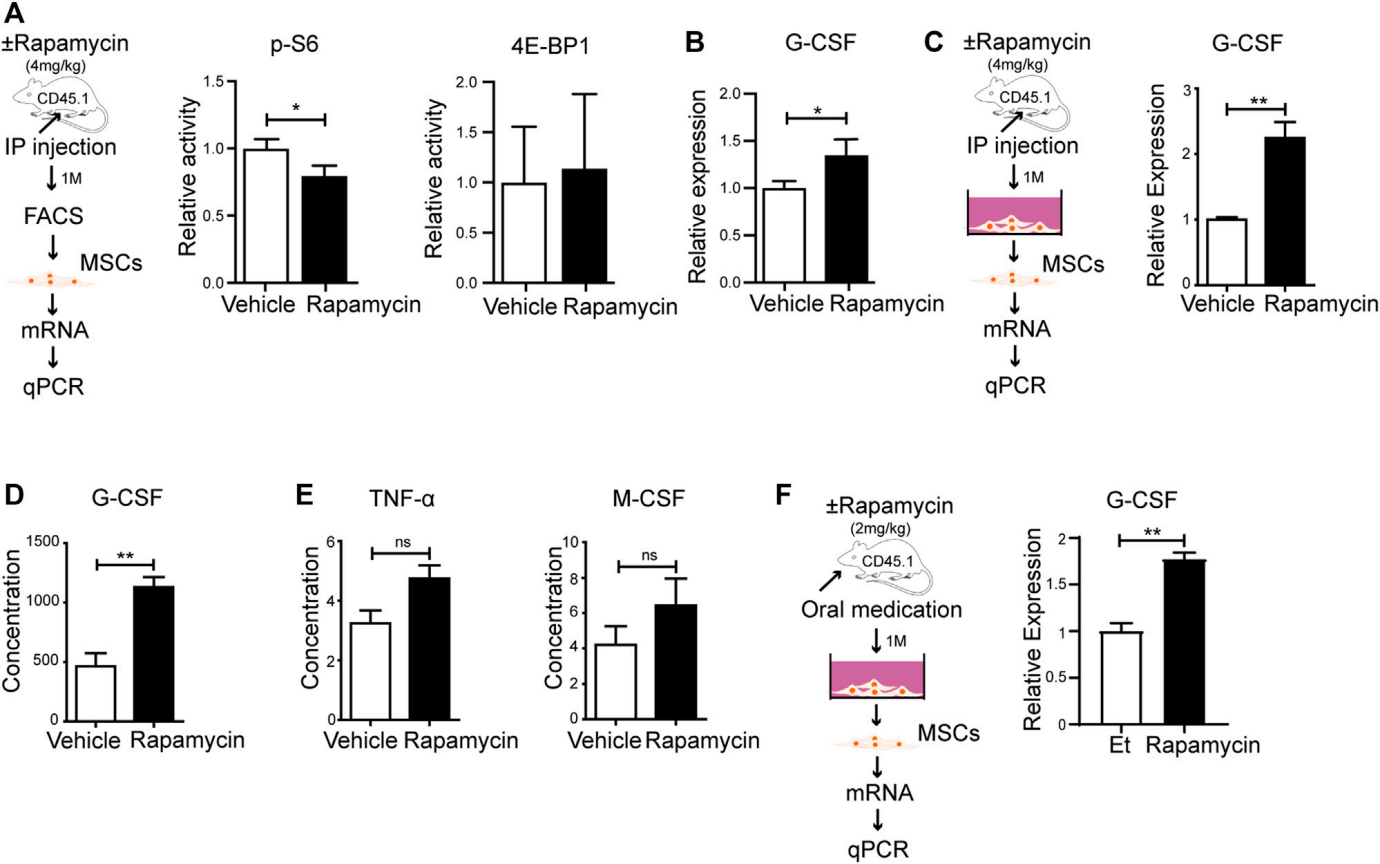
Rapamycin prompted MSCs to produce more G-CSF *in vivo* **(A)** Experiment design of MSCs isolated from mice treated with rapamycin or vehicle by i. p., n = 3, days = 30, dose = 4 mg/kg/day (left panel). Fluorescence intensity of p-S6 and p-4E-BP1 in MSCs treated by rapamycin or vehicle by i. p (right and middle panels) **(B)** mRNA relative expression of G-CSF in MSCs from mice treated with rapamycin or vehicle by i. p. **(C)** Experiment design of MSCs cultured *in vitro* (left panel). mRNA relative expression of G-CSF in MSCs after culture (right panel) **(D–E)** Relative expression of G-CSF、TNF- α and M-CSF in serum from mice treated with rapamycin or vehicle by Milliplex multifactor detection technology **(F)** Experiment design of MSCs isolated from mice treated with rapamycin or ethanol by p. o., n = 3, days = 30, dose = 2 mg/kg/day (left panel). Relative mRNA expression of G-CSF in MSCs of two groups (right panel).

To verify if rapamycin could increase the G-CSF expression in MSCs *in vivo*, we injected WT mice with rapamycin or vehicle by i. p. for 1 month. We sorted MSCs from the BM and analyzed phosphorylation level of S6 and 4 E-BP1 by FACS. We found that the phosphorylation of S6 was significantly reduced, while the phosphorylation of 4 E-BP1 was not changed in MSCs from mice treated with rapamycin when compared with that of controls (Figure 6A). The mRNA expression of G-CSF was increased in primary MSCs from mice treated with rapamycin when compared with that of controls (Figure 6B). Then, we cultured MSCs *in vitro* and analyzed G-CSF expression in MSCs by qPCR. We found that the G-CSF mRNA expression was significantly increased in MSCs from mice treated with rapamycin after culture when compared with that of controls (Figure 6C). In order to eliminate the immune response induced by rapamycin, we collected serum from mice treated with rapamycin or vehicle and measured the expression of inflammatory factors in serum. We found that the expression of G-CSF was significantly increased in serum of mice treated with rapamycin when compared with that of control mice (Figure 6D). However, TNF- α and M-CSF expression was not changed in serum of mice treated with rapamycin (Figure 6E). To assess the G-CSF expression in MSCs from mice treated with rapamycin or ethanol by p. o., MSCs were sorted and cultured *in vitro*. We found the G-CSF mRNA expression was significantly increased in MSCs from mice treated with rapamycin by p. o. when compared with that of controls (Figure 6F). These data indicated that rapamycin promote myeloid cells expansion through increasing G-CSF expression in MSCs.

## Discussion

The defects of bone marrow monocyte/macrophage differentiation caused by mTOR deficiency has been systematically studied with various mouse models. As a mTOR inhibitor, rapamycin could inhibit proliferation and impair differentiation of megakaryocyte and dendritic cell (Guo et al., 2013). In this study, by using murine models, we found that both intraperitoneal and oral administration of rapamycin led to remarkable expansion of myeloid lineage, which was caused by more G-CSF secreted by MSCs. Our data suggested that rapamycin influenced normal hematopoiesis by inhibiting mTOR signaling pathway.

Reducing mTORC1 activity was shown to lead to neutrophils immaturation (Wang et al., 2019). Interestingly, we found that intraperitoneal administration of rapamycin impaired neutrophil development, while oral administration of rapamycin did not influence the cells development. Since the absorption, distribution, and the circulating and/or tissue concentrations of rapamycin are subjected to the given dose and route (Magari et al., 1997), the degree of oral bioavailability and resultant circulating and/or tissue rapamycin concentration might be lower than intraperitoneal administration of rapamycin. It is conceivable that a higher dose of rapamycin treatment could completely inhibit mTOR. But a lower dose of rapamycin could potentially improve the tissue metabolic milieu by normalizing, and not completely inhibiting mTOR signaling (den Hartigh et al., 2018). Furthermore, this lower dose could also limit the complex feedback mechanisms involved in the activation of IRS1-PI3K-AKT. We found that p-S6 was reduced significantly in BM cells of recipient mice after treatment with rapamycin by p. o. or i. p. As a downstream target of mTORC1, S6K could negatively regulate mTORC2 (Malik et al., 2018), while mTORC2 activates AKT signaling pathway and promotes cell proliferation (Jacinto et al., 2006; Yang et al., 2006). The reduction in S6K phosphorylation/activation releases S6K’s inhibition of mTORC2, thus increases the phosphorylation level of AKT^S473^. This may cause an aberration in the S6K-mediated negative feedback loop which regulates mTORC2 activity, and further affects cell survival (Guo et al., 2013; Malik et al., 2018). Our findings suggested that oral administration of rapamycin by p. o. might have similar effects on proliferation of myeloid lineage cells when compared with the intraperitoneal administration of rapamycin by i. p., while oral administration of rapamycin by p. o. had little adverse effects on neutrophil differentiation through the feedback loop. Moreover, the oral administration of rapamycin might affect myeloid lineage cells proliferation by both mTORC1 and mTORC2 signaling pathways.

Rheb1 acts as a key upstream activator of mTOR to play vital roles in maintaining proper hematopoiesis and myeloid differentiation (Aspuria and Tamanoi 2004). Our previous studies found that the proliferation of myeloid lineage cells in BM and PB were increase in HSCs of *Vav1-Cre;Rheb1* ^*fl/fl*^ mice (Wang et al., 2019; Gao et al., 2021). In addition, mTOR-deficiency impaired GMPs differentiation into the monocyte/macrophage lineage (Zhao et al., 2018). In our data, the percentage of myeloid lineage cells was equivalent in BM and PB of *Rheb1* ^*fl/fl*^ mice and *Lyz-cre; Rheb1* ^*fl/fl*^ mice, in which Rheb1 was specifically deleted in monocytic lineage stage. Rheb1 deletion in mature monocytes/macrophages doesn’t affect the differentiation of myeloid lineage cells. Thus, we suspected that rapamycin might have influences on GMPs/HPCs/HSCs rather than the terminal monocytes/macrophages through mTORC1 signaling pathway.

In our study, we found the phosphorylation of S6 was significantly reduced in MSCs treated with rapamycin. It has been known that rapamycin could decrease the phosphorylation of S6, eIF4B and eEF2 by blocking the kinase activity of mTORC1, while it did not influence the expression of S6, eIF4B and eEF2 (Huo et al., 2011). So we think rapamycin affect MSCs by inhibiting the activity of mTORC1. The mechanisms by which mTOR inhibition protects from stem cell exhaustion and aging have been associated with secretion of major senescence-associated cytokines, such as SCF (Aspuria and Tamanoi 2004). In our studies, rapamycin significantly increased G-CSF expression in MSCs. G-CSF is secreted by bone marrow stromal cells, endothelial cells, macrophages, and fibroblast cells (Boettcher et al., 2014). G-CSF plays an important role in regulating immune cell number and function in allografts. Previous study indicated that G-CSF modulated NK subpopulations in PB and BM (Yu et al., 2018). G-CSF limited the IFN-γ signaling in T cells and induce immune tolerance (Franzke et al., 2003; Chang et al., 2019). In addition, G-CSF decreases the production of TNF-α, IL-2, IFN-γ, and modulates immune responses (Franzke et al., 2003). Moreover, G-CSF is the most important regulator that drives hematopoiesis of stem cells to differentiate into common myeloid progenitors and granulocyte/macrophage progenitors (Basu et al., 2002). Our studies indicate that rapamycin may promote the expansion of myeloid lineage cells in BM and PB through regulating G-CSF expression in MSCs by blocking mTORC1 signaling pathway, while the increased G-CSF in MSCs might be not robust enough to rescue the inhibition of myeloid cells differentiation induced by intraperitoneal administration of rapamycin *in vivo.*

## Data Availability

The original contributions presented in the study are included in the article/Supplementary Material, further inquiries can be directed to the corresponding author.

## Abbreviation

mTOR, mammalian target of rapamycin; 4E-BPI, 4E binding protein; S6, p70-kDa S6 ribosomal protein kinase; Rheb1^Δ/Δ^, Lyz-Cre; Rheb1^fl/fl^; MSC, Mesenchymal stem cells; G-CSF, Granulocyte-colony-stimulating factor.
